# The association between physical fitness, sports club participation and body mass index on health-related quality of life in primary school children from a socioeconomically deprived area of England

**DOI:** 10.1016/j.pmedr.2021.101557

**Published:** 2021-09-13

**Authors:** Laura Basterfield, Naomi L. Burn, Brook Galna, Guoda Karoblyte, Kathryn L. Weston

**Affiliations:** a Human Nutrition Research Centre and Population Health Sciences Institute, Newcastle University, Medical School, Framlington Place, Newcastle upon Tyne NE2 4HH, UK; b School of Health and Life Sciences, Teesside University, Constantine Building, Middlesbrough TS1 3BA, UK; c Health Futures Institute, Murdoch University, Perth, Australia; d Translational and Clinical Research Institute, Newcastle University, Newcastle upon Tyne NE2 4HH, UK; e School of Applied Sciences, Edinburgh Napier University, Sighthill Campus, Edinburgh EH11 4BN, UK

**Keywords:** Aerobic fitness, Quality of life, Child health, Socioeconomic status, Public health

## Abstract

We examined associations between physical fitness components, body mass index (BMI) and sports club participation on health-related quality of life (HRQoL) in 8- to 11-year-old children from a socioeconomically deprived region of England. From May-October 2019, 432 children completed the HRQoL questionnaire Kidscreen-27 and Leisure Time Physical Activity Survey, and a physical fitness testing battery of 20 m shuttle run test (20mSRT), handgrip strength (Handgrip), standing broad jump (Broad Jump), and sit-and-reach. Height, body mass, BMI and somatic maturity data were collected. Comparisons with reference populations were undertaken using a quintile framework. Linear and quantile regression assessed associations between physical fitness components and HRQoL variables. Using English Indices of Multiple Deprivation, 90% of children were from the most deprived quintile and 39% were overweight or obese. More children scored poorly on the Physical Wellbeing (40%) and Psychological Wellbeing (45%) HRQoL domains than the reference population (31%). Physical fitness scores were generally classed as ‘low’-’very low’ (42–58%). 20mSRT and Broad Jump performance explained an additional 10.7% of variance in Physical Wellbeing after adjusting for BMI z-score, sex and age (total R^2^ 21.2%). Quantile regression identified a subset of children who rated Physical Wellbeing as high regardless of 20mSRT performance. Sports club participation was associated with better 20mSRT and Broad Jump performance, and all domains of HRQoL. Our data indicate that some physical fitness components and sports club participation are positively associated with HRQoL of children from socioeconomically deprived areas, irrespective of BMI z-score. Interventions to improve HRQoL should consider both aspects.

## Highlights

• Children from a deprived region had inadequate physical fitness and excess weight.
• Children reported poor Physical Wellbeing and Psychological Wellbeing.
• Physical Wellbeing was related to 20 m Shuttle Run and Broad Jump performance.
• Effects of 20 m shuttle run performance on Physical Wellbeing were independent of BMI.
• Sport club attendance was positively associated with health-related quality of life.

## 1 Introduction

Health-related quality of life (HRQoL) is a complex, multidimensional concept, including social, emotional and physical functioning and well-being, related to the individual’s health state (Seid et al., 2000). In children, enhancing HRQoL is essential for their present and future well-being (Wallander and Koot, 2016) and widely considered a priority area for health interventions (Cartwright-Hatton et al., 2006, Ravens-Sieberer et al., 2014). Nonetheless, HRQoL appears to have declined in young people, with 10.3% of American adolescents reporting fair or poor health in the 2009–2010 National Health and Nutrition Examination Survey compared with 6.3% in 2001–2002 (Cui and Zack, 2013). Modifiable factors associated with HRQoL in children need identification, to develop appropriate public health interventions.

The relationship between physical activity and HRQoL in children is well documented (Marker et al., 2018). Systematic review evidence indicates higher levels of physical activity are associated with higher HRQoL scores in healthy child populations (Wu et al., 2017). More recently, the role of physical fitness and quality of life (QoL) outcomes in young people has also been explored. Distinct from physical activity, physical fitness can be defined as a set of characteristics related to health and performance, including aerobic fitness, muscular endurance, strength and power, body composition, flexibility, balance, agility, and reaction time (Caspersen et al., 1985, HOWLEY, 2001). Despite a global review calling for high quality studies into physical fitness, QoL and mental wellbeing in children (Lang et al., 2018), studies are limited, and none from England. Of those that have been conducted, associations between aspects of physical fitness and HRQoL may be apparent. For example, in n = 1158 Spanish 8–11-year-olds (Morales et al., 2013), greater Physical Wellbeing (using the Kidscreen-52 HRQoL questionnaire) was associated with better 20 m shuttle run test (20mSRT) performance and muscular fitness. In American 9–11-year-olds (n = 201) (Gu et al., 2016), positive associations between physical fitness and Physical Functioning were reported via the Pediatric Quality of Life Inventory (PedsQL) HRQoL questionnaire; and in Swiss 6–8-year-olds (n = 378) (Gerber et al., 2017), a weak positive association was observed between 20mSRT and the Physical Wellbeing and Psychological Wellbeing domains of the parent-reported KINDLR HRQoL questionnaire. Recently, the directionality of sports club participation and mental health outcomes in n = 3956 Australians aged 12–17 years was examined. Here, greater participation in team sport prospectively predicted fewer symptoms of depression and anxiety at subsequent timepoints (Graupensperger et al., 2021). While the heterogeneity of outcomes (e.g. depression, anxiety, quality of life) and populations (e.g. children, adolescents) across previous studies is acknowledged, the findings are promising from a mental wellbeing perspective.

It is also important to consider whether social inequalities impact on young peoples’ physical fitness and HRQoL. Previously, it was suggested that children from areas of lower socioeconomic status tend to have lower levels of physical activity (Love et al., 2019) and physical fitness (Wolfe et al., 2020) than their more affluent peers, and children living in more deprived areas are more likely to suffer from poor mental health (Mental Health Foundation, 2019). Accordingly, the aim of our study was to determine how 8–11-year-old children living in a relatively deprived region of England rated their HRQoL, and explore whether this was associated with physical fitness components (namely, 20mSRT, standing broad jump (Broad Jump), sit-and-reach performance and handgrip strength (Handgrip)), BMI and sports club participation.

## 2 Materials and methods

### 2.1 Ethics

The cross-sectional study was approved by Newcastle University Faculty of Medical Sciences Ethics Committee (approval number 1614/7165/2018) and conducted in accordance with the Declaration of Helsinki for experiments involving humans. Recruitment was undertaken by the Newcastle United Foundation (an independent registered charity).

### 2.2 Study participants

An academic trust covering four primary schools in connecting wards of Newcastle upon Tyne was approached; three schools responded and agreed to participate. Headteachers provided informed written consent for their school’s participation. Newcastle upon Tyne is a relatively deprived area, rated 74/317 local authorities in England, where 1 = most deprived (Ministry of Housing Communities & Local Government, 2019b). The consenting schools are located in areas that, by 2019 Index of Multiple Deprivation (Ministry of Housing Communities & Local Government, 2019b) ranking, are below the 0.1, 2nd and 14th centiles, respectively (where lower is more deprived). Parents/carers of Year 4 to 6 pupils (age 8–11-years, n = 469) received study information and the opportunity to opt-out their child. Six parents chose this option. Participating children were given verbal information and provided written or verbal assent to participate. There were no exclusion criteria. Data collection took place during the 2019 summer and autumn terms (May-July, and October 2019). Schools provided the sex, ethnicity, date of birth, home postcode of each child. Individual-level index of multiple deprivation (IMD) data were obtained using home postcode and freely-available IMD data (Ministry of Housing Communities, 2019a).

### 2.3 Measures

Physical fitness was assessed via the following age-appropriate, reliable, validated (Ruiz et al., 2011, Tomkinson et al., 2018) components of the Eurofit (EUROFIT, 1988) testing battery: 20mSRT, Handgrip, Broad Jump and sit-and-reach performance. Test procedures have been published previously (Weston et al., 2019), but briefly, measurements were as follows:

Height, seated height and weight were measured to the nearest 0.1 cm and 0.1 kg respectively, with the participant barefoot in light indoor clothing, using a portable stadiometer (Leicester Height Measure, SECA UK LtD, Birmingham, England) and calibrated scales (Shekel H151-7, Shekel Scales Ltd, Israel). Duplicate measures were taken and mean values used for analysis. Using the LMS Growth excel add-in, BMI and age- and sex-specific BMI z-scores relative to UK 1990 reference data (Cole et al., 1995) were calculated. Population-sensitive cut-points categorised children as either “underweight” (≤2nd centile or below), “healthy weight” (>2nd < 85th centile) “overweight” (≥85th < 95th centile) “obese” (≥95th < 99.6th centile) or “severely obese” (≥99.6th centile and above). Leg length was calculated by subtracting seated height from stature. Somatic maturity was estimated for each participant by predicting years from attainment of peak height velocity (PHV) via sex-specific equations (Mirwald et al., 2002). Participants were then classified as pre-peak or post-peak height velocity.

Aerobic fitness was indirectly assessed via 20mSRT performance using the British National Coaching Foundation protocol (Ramsbottom et al., 1988). Researchers ran alongside the children, encouraging them to run between cones 20 m apart in time to an audible bleep signal. Participants ran until they failed to maintain the pace for two consecutive bleeps, or at their own volition. Test performance was expressed as the total number of shuttles completed.

Handgrip was measured using a digital hand dynamometer (Grip-D, TKK 5401, Takei, Tokyo, Japan). Participants performed the test standing, with the wrist neutral and the elbow extended and arm not touching the body. Participants were instructed to squeeze the dynamometer as hard as they could for at least three seconds. Elbow flexion from 180⁰ to 90⁰ was permitted (Cohen et al., 2011). The test was repeated after a rest period of at least three minutes. Both hands were tested and dominant hand recorded. The maximum for both dominant and non-dominant hand was used in analysis.

Lower body power was estimated via standing broad jump (Broad Jump) performance. Participants stood behind a starting line with feet together and jumped forwards as far as possible. Three practice jumps were followed by three measured attempts. The distance jumped was measured from the back of the heel to the starting line using a tape measure. The maximum score was used in analysis.

Sit-and-reach performance was measured using a steel sit-and-reach box. Participants sat barefoot with their legs straight and feet against the box, then reached forward with both hands as far as possible. Three measured attempts followed three practices, recorded to the nearest 0.5 cm. The maximum score was used in analysis.

All physical fitness testing was conducted indoors, except for the 20mSRT in schools 2 and 3, due to lack of suitable indoor space. Testing outdoors took place on dry days with little wind, on a firm playing surface. Outdoor temperature varied from 10 to 19⁰C. There was no difference in mean number of shuttles run indoors and outdoors (24 v. 22 shuttles p = 0.217).

HRQoL was measured via the Kidscreen-27 (Ravens-Sieberer et al., 2006) questionnaire, validated to assess subjective health and wellbeing in children and adolescents aged 8–18 years. It has 27 items measuring five dimensions: Physical Wellbeing; Psychological Wellbeing, Parent Relations & Autonomy, Social Support & Peers; School Environment; at least 75% of items must be answered for a valid survey. Within each dimension, item scores are summed and transformed to T-scores with a mean ≈ 50 and SD ≈10. Higher scores indicate a higher HRQoL. Responses were considered ‘low’ if they were less than the mean-0.5*SD of the reference population (Ravens-Sieberer et al., 2006). Sports club participation was explored through the Leisure Time Physical Activity Survey (Burgess et al., 2006) (LTPAQ). Children provided details on whether they attended sports clubs at school and outside-school (i.e. in their leisure time), then type, weekly frequency and duration of club. Total time spent in sports clubs per week was calculated. Children completed the questionnaire by themselves if they were able to comprehend the questions, or as a class with the teacher reading each question. Assessors were trained by KW and LB, and worked in pairs. Measurement order was pragmatic, to fit with availability of children and testing space.

### 2.4 Statistical analysis

Data were analysed using SPSS v.24 (IBM) and R (R Core Team, 2020) (Quantreg package). To account for possible clustering due to the use of schools, a mixed model analysis with random effects was carried out, using school as the random effect variable, and physical fitness components/BMI z-score/HRQoL domains as dependent variables. The estimates of covariance parameters for school (as the intercept) were all p > 0.4, indicating that there was no clustering effect due to school. Data were therefore analysed as one group. Children were able to opt-out of elements of testing; missing data was not interpolated, analysis was performed on the data available.

Comparisons with published International and European fitness reference data (20mSRT, Handgrip, Broad Jump) were completed following recommendations (Tomkinson et al., 2017, Tomkinson et al., 2018). An age- and sex-specific quintile framework using the following centiles was adopted: <20th centile ‘very low’; ≥20^th^ < 40th centile ‘low’; ≥40^th^ < 60th centile ‘moderate’; ≥60^th^ < 80th centile ‘high’; ≥80^th^ centile ‘very high’. Exact scores corresponding to each quintile are available (De Miguel-Etayo et al., 2014, Tomkinson et al., 2017, Tomkinson et al., 2018). Reference protocols for sit-and-reach were substantially different to ours, so not used.

As most data were skewed, medians and interquartile range (IQR) are presented, and sex differences assessed using the independent samples median test. Associations between HRQoL domains, anthropometry (age/sex/BMI), and physical fitness components were assessed using forward stepwise linear regression. HRQoL domains were the dependent variables, confounders were added in block one (age, sex, BMI z-score), and physical fitness predictors in block two. Final models are presented. Age-to-PHV was initially added as a confounder, but due to high collinearity with age (VIF statistic > 10) and with <2% of the children post-PHV, this variable was omitted. Further examination of the data was performed using quantile regression, as this does not make assumptions about the distribution of the residuals. Chi^2^ tests were used for categorical variables. Significance was set at p < 0.05.

## 3 Results

Results are restricted to those who completed the Kidscreen-27 questionnaire (n = 432, 93% of potential population, 54% male). Missing questionnaires were due to absence or incomplete entries. Most children were from the White ethnic background (90.4%, with 4.2% Black, 2.6% Asian, 1.9% Mixed Ethnicity, 0.9% other ethnic background) and 90.0% of children were from the most deprived IMD quintile, followed by 5.1%, 4.6%, 0.0% and 0.2% in the second, third, fourth and fifth quintiles, respectively. School 1 contributed n = 108 children (25%), school 2 n = 87 children (20.1%), school 3 n = 237 children (54.9%). Of this total, n = 135 (31.3%) were in English Year 4 (age 8-9y), n = 144 (33.3%) Year 5 (age 9-10y), and n = 153 (35.4%) Year 6 (age 10-11y). One child of 12.0y was included.

Three children (0.7%) were classed as underweight relative to UK90 reference data, 245 (59.5%) were healthy weight, 55 (13.4%) overweight, 74 (18.0%) obese, and 34 (8.3%) severely obese. Twenty-one children did not have weight and/or height data due to absence or refusal. There were no statistically significant differences in BMI, BMI z-score or age between boys and girls, but the maturity estimates suggest that girls were closer to PHV (Table 1) and five were classed as post-PHV, whereas all boys were pre-PHV.

**Table 1:** Summary of unadjusted anthropometric, physical fitness components and health-related quality of life characteristics.

	Median (25th, 75th percentile)			
	All Children (n = 432*)	Boys (n = 232)	Girls (n = 200)	T-test (p)
*Anthropometry*				
Age (y)	9.9 (9.2, 10.7)	10.1 (9.3, 10.7)	9.7 (9.1, 10.7)	0.148
Age to peak height velocity (y)	−2.7 (-3.4, −2)	−3.3 (-3.8, −2.7)	−2 (-2.5, −1.3)	< 0.001
Post-peak height velocity (%)	1.2	0	2.5	–
BMI (kg.m^−2^)	18 (16.4, 21.2)	17.8 (16.2, 21.1)	18.5 (16.7, 21.2)	0.084
BMI z-score	0.71 (-0.11, 1.74)	0.73 (-0.19, 1.87)	0.67 (-0.1, 1.61)	0.516
*Physical fitness components*				
20 m shuttle run test (total shuttles run)	22 (15, 33)	25 (16, 39)	20 (14, 28)	0.001
Standing broad jump (maximum distance, cm)	128 (112, 144)	131 (118, 148)	122 (107, 135)	0.001
Sit-and-reach (maximum reach, cm)	16 (10, 21)	14 (8, 18)	18 (12, 23)	<0.001
Handgrip strength - dominant hand (maximum, kg)	15.3 (12.4, 17.4)	15.7 (12.6, 18.1)	14.6 (12.2, 16.6)	0.012
Handgrip strength - non-dominant hand (maximum, kg)	14.5 (11.9,17.3)	15.3 (12.0, 17.6)	13.8 (11.9, 16.7)	0.017
*Health-related quality of life domain (T scores)*				
Physical Wellbeing	49.6 (44.7, 59.4)	52.4 (44.7, 59.4)	47.1 (42.5, 55.6)	0.054
Psychological Wellbeing	50.6 (43.2, 56)	50.6 (43.2, 56)	50.6 (43.2, 56)	0.406
Autonomy & Parents	49.5 (44, 59.1)	49.5 (44, 59.1)	49.5 (44, 59.1)	0.699
Social Support & Peers	53.2 (44.4, 66.3)	57.8 (44.4, 66.3)	52 (44.4, 66.3)	0.063
School Environment	54.4 (45.4, 62.8)	54.4 (45.4, 62.8)	54.4 (45.4, 62.8)	0.416

* Individual n will vary due to missing data.

There were sex differences for all physical fitness variables (Table 1); boys ran more shuttles in the 20mSRT (25 v. 20, p = 0.001), jumped further (131 cm v. 122 cm, p = 0.001), and had greater Handgrip than girls. Sit-and-reach performance was significantly greater in girls than boys (18 cm v. 14 cm p < 0.001). When compared against International and European reference populations (Table 2), 58% of children scored in the ‘very low’ or ‘low’ categories for the 20mSRT, 41.7% for Handgrip and 56.5% for Broad Jump. Percentages of children in each category were generally weighted towards very low/low rather than high/very high (Table 2).

**Table 2:** Comparison of physical fitness components against International (Tomkinson et al., 2017) and European (De Miguel-Etayo et al., 2014, Tomkinson et al., 2018) reference standards using a quintile framework.

Quintile	Physical fitness variable (% of children in each category)		
	Total shuttles (n = 407)	Handgrip strength (n = 424)	Standing broad jump (n = 414)
Very low	32.3	17.7	30.2
Low	25.8	24.1	26.3
Moderate	13.0	25.5	14.7
High	15.5	20.8	16.2
Very high	13.5	12.0	12.6

N.B. Reference data for sit-and-reach with similar protocol were not available.

Participants’ HRQoL scores are in Table 1, and median values were broadly in line with the reference population (Ravens-Sieberer et al., 2007). As distributions were skewed due to children with high scores, we also investigated whether more children were reporting low scores. For Physical Wellbeing and Psychological Wellbeing, 40.4% and 45.3% of children were classed as ‘Low’, greater than the expected 31% of the reference population (Ravens-Sieberer et al., 2007). BMI z-score (after adjusting for age and sex) was negatively associated with Physical Wellbeing (β-1.78, 95% CI −2.61,-0.95 p < 0.001) and Psychological Wellbeing (β-1.05, −1.88,-0.22 p = 0.014). There was no association with the other HRQoL domains.

Bivariate correlations between descriptive and physical fitness predictors, and HRQoL domains before and after adjusting for age, sex and BMI are in Appendix A.

Regression analyses between physical fitness variables and HRQoL are in Table 3A. For Physical Wellbeing, age/sex/BMI z-score alone accounted for nearly 11% of variability, however the fully adjusted model including both Broad Jump and 20mSRT explained 21.2% of the variation in Physical Wellbeing. Small but significant associations were found for the other HRQoL domains (Table 3A).

**Table 3:** Summary of linear regression models predicting health-related quality of life by: A) physical fitness and B) sports club participation.

	Health-Related Quality of Life (T-scores) Unstandardised B (CI 95%)				
	Physical Wellbeing	Psychological Wellbeing	Autonomy & Parents	Social Support & Peers	School Environment
*A Block 1 predictors (forced enter)*					
Constant	55.8 (43.9, 67.6) p < 0.001	47.8 (35.5, 60.0) p < 0.001	22.0 (8.2, 35.8) p = 0.002	45.4 (31.82, 58.92) p < 0.001	46.4 (33.77, 59.02) p < 0.001
Sex (Female)	−1.66 (-3.77, 0.46) p = 0.124	−1.27 (-3.44, 0.89) p = 0.247	0.56 (-1.85, 2.96) p = 0.650	−0.61 (-3.02, 1.8) p = 0.619	0.8 (-1.43, 3.04) p = 0.480
Age (years)	−2.18 (-3.31, −1.05) p < 0.001	0.57 (-0.59, 1.72) p = 0.333	1.95 (0.63, 3.27) p = 0.004	−0.03 (-1.48, 1.41) p = 0.966	0.62 (-0.57, 1.8) p = 0.305
BMI z-score	−0.12 (-1.05, 0.82) p = 0.803	−0.84 (-1.72, 0.03) p = 0.058	0.36 (-0.64, 1.36) p = 0.476	−1.27 (-2.33, −0.21) p = 0.020	−0.03 (-0.92, 0.87) p = 0.956
*Block 2 with physical fitness predictors (forward stepwise)*					
20mSRT	0.19 (0.09, 0.29) p < 0.001	–	–	–	–
Standing broad jump (cm)	0.12 (0.07, 0.17) p < 0.001	–	0.07 (0.02, 0.13) p = 0.010	–	–
Sit-and-reach (cm)	–	–	–	–	–
Dominant hand grip strength (kg)	–	–	–	0.69 (0.3, 1.09) p = 0.001	–
*Model Summary*					
Final model adjusted (R^2^)	21.2%	0.8%	5.0%	4.7%	<1%
Change with Block 2 (ΔR^2^; p)*	10.7% (p < 0.001)	1.6% (p = 0.106)	1.8% (p = 0.010)	3.2% (p = 0.001)	<1% (p = 0.707)
*B Block 1 predictors (force enter)*					
Constant	62.4 (51.1, 73.6) p < 0.001	48.1 (36.5, 59.8) p < 0.001	25.4 (12.7, 38.2) p < 0.001	41.8 (28.6, 54.9) p < 0.001	46.6 (34.5, 58.7) p < 0.001
Sex (Female)	−3.43 (-5.44, −1.42) p = 0.001	−0.93 (-3, 1.13) p = 0.373	0.79 (-1.47, 3.05) p = 0.493	−0.72 (-3.06, 1.61) p = 0.544	0.78 (-1.36, 2.92) p = 0.474
Age (y)	−0.75 (-1.81, 0.3) p = 0.161	0.29 (-0.8, 1.38) p = 0.600	2.36 (1.17, 3.55) p < 0.001	1.16 (-0.07, 2.39) p = 0.064	0.48 (-0.65, 1.6) p = 0.407
BMI z-score	−1.7 (-2.5, −0.9) p < 0.001	−0.99 (-1.81, −0.17) p = 0.019	−0.04 (-0.94, 0.87) p = 0.934	−0.32 (-1.25, 0.62) p = 0.505	−0.09 (-0.94, 0.77) p = 0.841
Block 2 with sport club participation**(force enter)					
School sport club	1.52 (-0.56, 3.59) p = 0.152	1.15 (-0.99, 3.28) p = 0.291	1.03 (-1.31, 3.37) p = 0.389	1.24 (-1.19, 3.66) p = 0.316	1.76 (-0.46, 3.98) p = 0.120
Outside-school club	5.69 (3.65, 7.73) p < 0.001	2.99 (0.89, 5.08) p = 0.005	3.2 (0.91, 5.49) p = 0.006	2.64 (0.26, 5.01) p = 0.030	1.62 (-0.56, 3.8) p = 0.145
*Model Summary*					
Final model adjusted (R^2^)	14.1%	3.2%	4.7%	1.7%	<1%
Change with Block 2 (ΔR^2^; p)*	7.9% (p < 0.001)	2.5% (p = 0.005)	2.3% (p = 0.008)	1.7% (p = 0.030)	1.7% (p < 0.056)

BMI = Body Mass Index; 20mSRT = 20 m shuttle run test, * ΔR^2^ is not adjusted for degrees of freedom and so may be greater than adjusted R^2^

** sport participation included as a binary variable (participation or not).

**Fig. 1:**
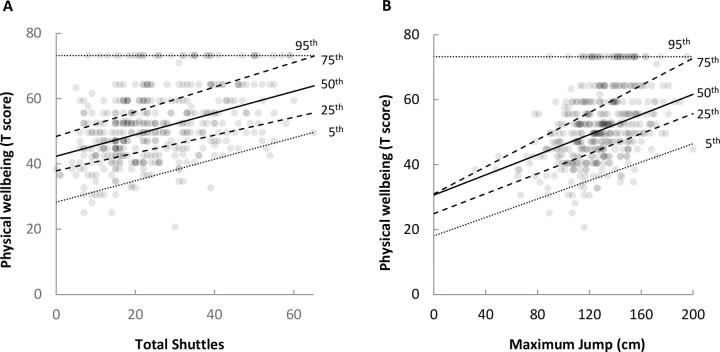
Quantile (5th, 25th, 50th, 75th, 95th) regression illustrating better 20mSRT (total shuttles) and Broad Jump (maximum jump distance) are associated with better Physical Wellbeing except for those with the best Physical Wellbeing for their physical fitness (95th quantile).

The relationship between Physical Wellbeing and 20mSRT and Broad Jump was generally linear throughout most of the distribution (children with a higher 20mSRT and Broad Jump performances consistently rated their Physical Wellbeing as high), however there was some heteroscedastic variance, (Fig. 1A and 1B). Quantile regression showed that the slopes of the lines across the 5th, 25th, 50th and 75th percentiles were relatively consistent, but the 95th percentile demonstrated a group that rated their Physical Wellbeing highly, regardless of their performance in the 20mSRT/Broad Jump.

Of the 432 children, 81 boys and 85 girls took part in at least one school-sports club, whereas 111 boys and 77 girls participated in at least one outside-school sports club. Time spent in sports clubs per week ranged from 30 to 360 min for school-sports, and 30–1200 min for outside-school sports. For analysis, times were capped at 600 min as this included time up to the 95th centile. There was no association between time spent in sport participation and BMI or BMI z-score, but linear regression (Appendix B) showed positive associations between total time spent in sports clubs each week, and 20mSRT and Broad Jump. Χ^2^ tests showed outside-school sports were associated with higher quintiles for 20mSRT (p < 0.001) and Broad Jump (p < 0.001), but not Handgrip (p = 0.584).

Children who participated in sports clubs had a higher HRQoL than non-participants (Appendix C), with school-sports clubs positively associated with Social Support & Peers, and School Environment, and outside-school sports clubs positively associated with all but School Environment. Linear regression using binary variables for sports participation against HRQoL showed small but significant positive associations for outside-school sport participation with all HRQoL domains except School Environment, even when accounting for age/sex/BMI (Table 3B). Together, these variables explained 14.1% of the variance in Physical Wellbeing, with sport participation contributing 7.9%. This figure varied between 1.7% and 2.5% for the other HRQoL domains.

## 4 Discussion

Despite evidence of a relationship between physical fitness and markers of mental wellbeing in adolescents (Åvitsland et al., 2020, Eddolls et al., 2018), associations between physical fitness, BMI and HRQoL in primary school-aged children are under-researched. We therefore aimed to determine how 8- to 11-year-old children living in a relatively deprived region of England rated their HRQoL and explore whether this was associated with physical fitness, BMI and sports club participation.

In our sample, median scores of five HRQoL dimensions were broadly in line with normative data (Ravens-Sieberer et al., 2006) . However, for Physical Wellbeing and Psychological Wellbeing, a greater than expected proportion of children were classed as ‘low’. This could be reflective of the low SES of our participants (Poulain et al., 2019), as deprivation is known to causally affect children’s outcomes, including physical and mental health (Cooper and Stewart, 2017), and adult HRQoL is predicted by deprivation (Kashem et al., 2019). Our data also suggests that interventions to improve HRQoL in this population may be required. When our participants’ physical fitness scores were compared to International and European reference populations using a quintile framework, children were not evenly distributed across quintiles, but were generally clustered around the lower categories. Below the 20th centile (‘very low’) has been suggested as the cut-off for being classed as ‘unfit’ (Tomkinson et al., 2018), meaning ∼ 33% of our sample were unfit for 20mSRT and Broad Jump but not Handgrip (17.7%). Our previous work with similarly-aged children in Gateshead (Weston et al., 2019), North East England, 9 miles from the current study location, reported higher 20mSRT than the current study (31 shuttles run compared with 22), with fewer children in the ‘very low’ quintile for 20mSRT (12% v. 32%). The previous study had more healthy weight children (70% v. 60%), but more children in the ‘very low’ Handgrip quintile (29% v. 18%) (Weston et al., 2019). This demonstrates the need to sample extensively, and that interventions aiming to improve physical fitness in this population may be required.

When exploring the relationship between HRQoL and physical fitness, 20mSRT and Broad Jump collectively explained 10.7% of the variability of Physical Wellbeing. While differences in HRQoL assessment tools render between-study comparisons problematic, this contribution is similar to cross-sectional data of 201 9–11-year-olds from the United States, where physical fitness (composed of aerobic fitness, muscular fitness, flexibility and BMI) explained 11.8% of the variability in the ‘physical functioning’ domain of the PedsQL questionnaire (Gu et al., 2016) (the addition of age/sex/BMI z score further increased our R^2^ to 21.2%). In our study, BMI contributed less to HRQoL than physical fitness, confirming that reported by Gu et al., (Gu et al., 2016), which is important since approximately 40% of children in our sample were overweight/obese, higher than both English and regional averages (Public Health England, 2019). Although analysed differently, Morales et al. used the longer Kidscreen-52 (Morales et al., 2013), and cardiorespiratory fitness (20mSRT performance categorized as poor/satisfactory/good) predicted Physical Wellbeing scores when controlling for age, muscular fitness (calculated using z-scores for both Handgrip and Broad Jump/body mass) and BMI in girls only (β 0.163, p < 0.001, similar to our 0.19 for 20mSRT). Muscular fitness was positively associated with Physical Wellbeing in both sexes (boys β 0.191p = 0.001, girls 0.194, p < 0.001, no R^2^ reported; we found 0.12 for Broad Jump) (Morales et al., 2013). Again, BMI had less influence on HRQoL than fitness.

While the relationship between 20mSRT and Broad Jump and Physical Wellbeing was generally linear throughout most of our distribution, quantile regression showed that the 95th percentile demonstrated a group that rated their Physical Wellbeing highly, regardless of their 20mSRT and Broad Jump performance. While these children tended to be younger and HRQoL decreases as children get older (Michel et al., 2009), the exact reasons for this finding remain unclear. Further study of these children is needed to consider other factors like perception-ability mismatch, since younger children tend to over-estimate their physical abilities (Washburn and Kolen, 2018), and may be unaware their performance was poor, whilst also over-estimating their physical health.

We observed statistically significant relationships between Broad Jump and Autonomy & Parents, and between Handgrip and Social Support & Peers, however, these fitness variables explained a small amount of variability in HRQoL (R^2^ = 2.3% and 1.7%, respectively). There are several potential mechanisms in children and adolescents whereby physical fitness may affect mental wellbeing, including neurobiological (i.e. changes in the structural and functional composition of the brain (Belcher et al., 2021)), neuroendocrinological (i.e. enhanced neuroendocrine responses to stressors (Silverman and Deuster, 2014)) and psychosocial mechanisms (i.e social interaction and self-efficacy (Lubans et al., 2016)). While these mechanisms are unconfirmed, establishing the directionality of the link between physical fitness and mental wellbeing is crucial for solidifying policy that promotes fitness and wellbeing at an early age.

Participation in sports clubs was positively associated with 20mSRT and Broad Jump, but also with HRQoL. This included small but significant effects in domains of HRQoL not associated with physical fitness. Possible reasons for these associations requires exploration as potential targets for interventions aiming to improve HRQoL, as the social benefits of peers and role models could be making important contributions to HRQoL. Vella et al., (Vella et al., 2014) conducted a large survey (n = 4042) on children’s sports club participation, and reported positive relationships with HRQoL, particularly in the Physical domain, where continued participation from age 8y to 10y was associated with an increase of 5 points in parent-reported PedsQL (Vella et al., 2014). Further, Moeijes et al. (Moeijes et al., 2019) used the Kidscreen-52 questionnaire with 1876 10–12-year-olds, and reported a significant association between sports participation and Physical Wellbeing of similar magnitude to that we reported (unstandardised beta coefficient 4.78 v. 5.69), as well as associations with the other HRQoL domains except School Environment, which we also demonstrated.

We successfully collected contemporary and comprehensive data, including individual-level deprivation, from a relatively deprived population who may be most at risk of future health problems (Kivimäki et al., 2020). However, generalisation of our findings to more affluent areas may be difficult. Our participants were predominantly from a white ethnic background, reflecting the geographic region, but not necessarily applicable to other ethnicities. Given the cross-sectional nature of this study, we are unable to infer causality between physical fitness and HRQoL, but longitudinal follow-up will help to address this.

## 5 Conclusions

We have demonstrated that children from a deprived part of England scored poorly on two of five domains of HRQoL, (Physical Wellbeing and Psychological Wellbeing), had inadequate physical fitness, and a large proportion were overweight. Future interventions seeking to improve HRQoL should consider both physical health and social angles, as collectively these aspects may be most impactful on populations in greatest need.

## Funding source

This study was helped by funding from the North of England Commissioning Support Unit.

## CRediT authorship contribution statement

**Laura Basterfield:** Conceptualization, Formal analysis, Investigation, Writing - original draft, Supervision. **Naomi L. Burn:** Investigation, Writing - original draft. **Brook Galna:** Formal analysis, Writing - review & editing. **Guoda Karoblyte:** Investigation, Writing - review & editing. **Kathryn L. Weston:** Conceptualization, Writing - original draft, Supervision.

## Declaration of Competing Interest

The authors declare that they have no known competing financial interests or personal relationships that could have appeared to influence the work reported in this paper.
