# Supplementary material for: The association between physical fitness, sports club participation and body mass index on health-related quality of life in primary school children from a socioeconomically deprived area of England
Source: Prev Med Rep. 2021 Sep 13;24:101557. doi: 10.1016/j.pmedr.2021.101557 (PMC8683957; doi:10.1016/j.pmedr.2021.101557)
Supplement: Supplementary data 1 [file mmc1.docx]

Appendices

Appendix A

Supplementary Table 1. Bivariate correlations between descriptive and physical fitness predictors, and Health-Related Quality of Life domains before and after adjusting for age, sex and BMI.

| **Variable** | **Descriptives** | |  | **Physical Fitness** | | | |
| --- | --- | --- | --- | --- | --- | --- | --- |
|  | **Age (y)** | **BMI z-score** |  | **20m shuttle run test (total shuttles)** | **Standing broad jump (cm)** | **Ssit and reach (cm)** | **Dominant handgrip strength (kg)** |
| **Unadjusted Pearson's R (p)** |  |  |  |  |  |  |  |
| ***Descriptives*** |  |  |  |  |  |  |  |
| Age (y) | - | .003 (.959) |  | ***.179 (<.001)*** | ***.281 (<.001)*** | ***-.125 (.010)*** | ***.451 (<.001)*** |
| BMI z-score | .003 (.959) | - |  | ***-.458 (<.001)*** | ***-.265 (<.001)*** | -.079 (.112) | ***.377 (<.001)*** |
| ***Physical Fitness*** |  |  |  |  |  |  |  |
| Total shuttles | ***.179 (<.001)*** | ***-.458 (<.001)*** |  | - | ***.594 (<.001)*** | .086 (.087) | ***.150 (.003)*** |
| Standing broad jump (cm) | ***.281 (<.001)*** | ***-.265 (<.001)*** |  | ***.594 (<.001)*** | - | ***.251 (<.001)*** | ***.380 (<.001)*** |
| Sit and reach (cm) | ***-.125 (.010)*** | -.079 (.112) |  | .086 (.087) | ***.251 (<.001)*** | - | .058 (.234) |
| Dominant handgrip strength (kg) | ***.451 (<.001)*** | ***.377 (<.001)*** |  | ***.150 (.003)*** | ***.380 (<.001)*** | .058 (.234) | - |
| ***Health Related quality of Life (T-scores)*** | |  |  |  |  |  |  |
| Physical wellbeing | -.065 (.183) | ***-.196 (<.001)*** |  | ***.383 (<.001)*** | ***.387 (<.001)*** | ***.153 (.002)*** | .022 (.655) |
| Psychological wellbeing | .018 (.709) | ***-.121 (.015)*** |  | ***.121 (.015)*** | ***.160 (.001)*** | .022 (.653) | .029 (.550) |
| Autonomy & parents | ***.185 (<.001)*** | -.010 (.841) |  | .086 (.086) | ***.184 (<.001)*** | ***.098 (.045)*** | ***.163 (.001)*** |
| Social support & peers | .080 (.097) | -.037 (.460) |  | ***.172 (.001)*** | ***.180 (<.001)*** | .086 (.079) | ***.143 (.003)*** |
| School | .036 (.459) | -.016 (.746) |  | .031 (.532) | .041 (.404) | .040 (.414) | .044 (.374) |
|  |  |  |  |  |  |  |  |
| **Partial R (p) after controlling for age, sex and BMI(z-score)** | | |  |  |  |  |  |
| ***Health Related quality of Life (T-scores)*** | |  |  |  |  |  |  |
| Physical wellbeing |  |  |  | ***.320 (<.001)*** | ***.333 (<.001)*** | ***.184 (.001)*** | ***.141 (.008)*** |
| Psychological wellbeing |  |  |  | .036 (.497) | .090 (.093) | .011 (.830) | .085 (.113) |
| Autonomy & parents |  |  |  | .082 (.127) | ***.135 (.011)*** | .103 (.054) | ***.133 (.013)*** |
| Social support & peers |  |  |  | ***.146 (.006)*** | ***.131 (.014)*** | ***.118 (.028)*** | ***.182 (.001)*** |
| School |  |  |  | .033 (.543) | .029 (.591) | -.015 (.783) | .049 (.357) |

BMI: Body Mass Index; Correlations with p < 0.05 appear in bold and italics

Appendix B

Supplementary Table 2. Linear regression model predicting 20m shuttle run test and standing broad jump by total time spent in sports clubs, and sex

| **Independent variables:** | **B coefficient** | **95% confidence interval for B** | **Adjusted R^2^** | **p** |
| --- | --- | --- | --- | --- |
| **Dependent variable:** 20mShuttle run test (total shuttles run) | | | | |
| Total time spent in sports clubs (min) | 0.020 | 0.008 to 0.032 | 0.111 | 0.001 |
| Sex | -6.941 | -10.286 to -3.595 |  | <0.001 |
| **Dependent variable:** Jump distance (cm) | | | | |
| Total time spent in sports clubs (min) | 0.035 | 0.013 to 0.057 | 0.068 | 0.002 |
| Sex | -8.316 | -14.334 to -2.297 |  | 0.007 |

Appendix C

Supplementary Table 3. Anthropometry, fitness and health-related quality of life (HRQoL) variables, by sports participation

|  | **School sports club** | | | | | **Outside-school sports club** | | | | | |
| --- | --- | --- | --- | --- | --- | --- | --- | --- | --- | --- | --- |
|  | **Participant (n=166)** | | **Non-participant (n=266)** | |  | **Participant (n=188)** | | **Non-participant (n=244)** | |  |  |
| **Variable** | **Median** | **25^th^, 75^th^ quartile** | **Median** | **25^th^, 75^th^ quartile** | **p** | **Median** | **25^th^, 75^th^ quartile** | **Median** | **25^th^, 75^th^ quartile** | **p** |  |
| **Anthropometry** | | | | | | | | | | | |
| BMI | 18.0 | 16.3,21.0 | 18.1 | 16.4, 21.2 | 0.950 | 17.9 | 16.3, 21.1 | 18.2 | 16.4,21.5 | 0.425 |  |
| BMI z-score | 0.60 | -0.18 | 0.79 | -0.06,1.78 | 0.201 | 0.67 | -0.22,1.66 | 0.73 | -0.05, 1.76 | 0.842 |  |
| **Fitness** | | | | | | | | | | | |
| 20m shuttle run test, total shuttles run | 25 | 16, 38 | 21 | 15,31 | 0.005 | 27 | 17,40 | 19 | 14,28 | <0.001 |  |
| Standing broad jump (cm) | 130 | 116, 149 | 125 | 110,140 | 0.052 | 135 | 119,150 | 122 | 108,135 | <0.001 |  |
| Sit and reach (cm) | 17.0 | 10.6,21.0 | 15.0 | 9.4,20.0 | 0.105 | 17.0 | 11.4,20.6 | 14.5 | 9.0,20.5 | 0.062 |  |
| Dominant handgrip strength (kg) | 15.4 | 12.9,17.6 | 15.0 | 12.1,17.2 | 0.320 | 15.3 | 12.5,17.7 | 15.1 | 12.2,17.1 | 0.672 |  |
| Non-dominant handgrip strength (kg) | 15.4 | 12.5,17.6 | 14.1 | 11.7, 17.1 | 0.033 | 14.8 | 11.9,17.3 | 14.1 | 11.8,17.3 | 0.202 |  |
| **HRQoL domain** | | | | | | | | | | | |
| Physical wellbeing | 52.4 | 44.7,59.4 | 49.6 | 42.5,55.6 | 0.239 | 52.4 | 47.1,64.3 | 47.1 | 41.5, 55.6 | <0.001 |  |
| Psychological wellbeing | 50.6 | 44.8,59.5 | 48.5 | 42.8,56.0 | 0.463 | 50.6 | 44.8,59.5 | 48.5 | 41.8, 53.1 | 0.001 |  |
| Autonomy & parents | 51.2 | 45.2,59.1 | 47.9 | 42.9,59.1 | 0.101 | 51.2 | 45.6,62.8 | 47.9 | 42.9,55.8 | 0.011 |  |
| Social support & peers | 57.8 | 46.9,66.3 | 53.2 | 44.4,66.3 | 0.092 | 57.8 | 46.9,66.3 | 53.2 | 44.4,66.3 | 0.004 |  |
| School environment | 56.3 | 48.1,62.8 | 51.1 | 45.4,62.8 | 0.038 | 54.4 | 48.1,62.8 | 51.1 | 45.4,62.8 | 0.440 |  |

BMI: Body Mass Index
